# Transthoracic signs of left atrial cardiomyopathy predict presence of left atrial thromboembolic substrate

**DOI:** 10.1016/j.hroo.2025.12.016

**Published:** 2026-01-07

**Authors:** Athanasios Frydas, Alice Sokour, Fabian Spinka, Victor Schweiger, Hong Ran, Florian Blaschke, Henryk Dreger, Leif-Hendrik Boldt, Abdul Shokor Parwani, Gerhard Hindricks, Daniel-Armando Morris, Ingo Hilgendorf, Matthias Schneider-Reigbert

**Affiliations:** 1 Deutsches Herzzentrum der Charité, Department of Cardiology, Angiology and Intensive Care Medicine, Berlin, Germany; 2 Charité – Universitätsmedizin Berlin, corporate member of Freie Universität Berlin and Humboldt-Universität zu Berlin, Berlin, Germany; 3 DZHK (German Center for Cardiovascular Research) partner site Berlin, Germany; 4 Nanjing First Hospital, Nanjing, China

**Keywords:** Left atrial strain, Spontaneous echo contrast, Thrombus, Atrial fibrillation, Transesophageal echocardiography, Thromboembolic risk

## Abstract

**Background:**

Transthoracic echocardiographic (TTE) parameters of left atrial (LA) size and function are independent risk factors for cardioembolic events. Nevertheless, they are not included in current decision trees evaluating the necessity of transesophageal echocardiography (TOE) before rhythm control.

**Objective:**

To evaluate whether TTE-assessed LA cardiomyopathy predicts LA thromboembolic substrate (LATS), defined as spontaneous echo contrast (SEC) ≥2+, sludge, or thrombus in the LA appendage (LAA).

**Methods:**

This retrospective study included 645 patients undergoing same-session TTE and TOE; 384 in sinus rhythm (SR) and 261 with atrial fibrillation/flutter (AF/AFL). A low-risk subgroup (n = 323) was defined per guideline criteria and were categorized as SR with prior AF (n = 105), AF/AFL (n = 157), or controls without AF (n = 61). TTE images regarding LA size and function were analyzed and correlated with TOE findings of LATS.

**Results:**

LA thromboembolic substrate was present in 59/323 (18.3%) low-risk patients, including 54/157 (34.4%) of those with AF/AFL. Among low-risk AF/AFL patients, 8.9% had LAA sludge or thrombus. Multivariable analysis showed only LA strain (LAS) independently predicted LATS; each 1% increase in LAS was associated with an 18% reduction in the odds of LATS (odds ratio 0.819, *P <* .001). Model discrimination improved markedly once LAS was added to a clinical-only model (area under the curve 0.86 vs 0.91, *P <* .001).

**Conclusion:**

LAS was the most accurate TTE parameter for identifying LATS, indicating that reliance solely on clinical risk scores may miss high-risk patients. Assessment of LA cardiomyopathy by TTE could guide individualized decisions regarding TOE before elective rhythm-control procedures.

## Graphical abstract

**Figure:**
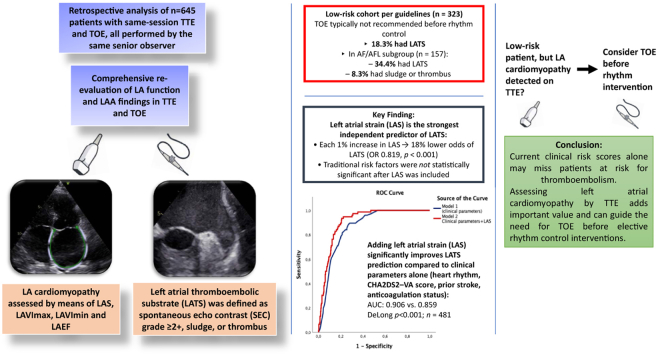

**Key Findings**

▪ Left atrial strain (LAS) was the only independent transthoracic echocardiographic (TTE) predictor of left atrial thromboembolic substrate (LATS); each 1% increase in LAS was associated with an 18% reduction in the odds of LATS (odds ratio 0.819, *P* < .001).
▪ Adding LAS to a clinical-only model significantly improved discrimination for LATS (area under the curve 0.91 vs 0.86, *P* < .001).
▪ In the low-risk atrial fibrillation/flutter cohort, 34.4% had LATS and 8.9% had sludge or thrombus, despite meeting guideline-based low-risk criteria.
▪ LAS demonstrated superior diagnostic accuracy compared with conventional left atrial volumetric parameters (left atrial volume index max, left atrial volume index min, left atrial emptying fraction) across all rhythm categories.
▪ Assessment of left atrial cardiomyopathy via TTE could improve patient selection for transesophageal echocardiography before elective rhythm-control procedures.

## Introduction

Current guidelines of the European Society of Cardiology allow omitting transesophageal echocardiography (TOE) to exclude left atrial appendage (LAA) thrombus prior to electrical cardioversion or catheter ablation in patients deemed low-risk, defined as those with adequate anticoagulation for at least 3 weeks. Exceptions include uncertain adherence, prior LAA thrombus, moderate or severe mitral stenosis (MS), or mitral valve surgery for MS, cardiac amyloidosis, hypertrophic cardiomyopathy, and history of stroke or transient ischemic attack (TIA). TOE is also not required in patients in sinus rhythm (SR) without anticoagulation if Congestive heart failure, Hypertension, Age ≥75 years [2 points], Diabetes mellitus, prior Stroke or TIA [2 points], Vascular disease, Age 65–74 years) equals 0 (CHA_2_DS_2_-VA).^1^^,^^2^

LA cardiomyopathy (LA-CMP) is defined as a combination of anatomical, structural, and functional alterations and can be diagnosed via transthoracic echocardiography (TTE). It commonly occurs secondary to left heart valve disease and/or left ventricular systolic and diastolic dysfunction. However, it can also manifest as a unique pathology. Its presence is a well-established risk factor for cardioembolic stroke.^3^^,^^4^ Among echocardiographic markers of LA function, LA strain (LAS) has been shown to be a sensitive and reproducible parameter that reflects the degree of LA remodeling and dysfunction, and is often reduced in patients with atrial fibrillation (AF) and heart failure (HF).^5^^,^^6^ Moreover, LAS has been shown to be better correlated with the degree of LA dysfunction compared with the most widely used LA volume index (LAVI), which is a purely volumetric parameter.^7^^,^^8^

Despite the proven association of LA-CMP and LA thromboembolic substrate (LATS), when addressing the risk of cardioembolic stroke before cardioversion or ablation therapy, current risk stratification models rely primarily on clinical parameters, and do not incorporate echocardiographic parameters regarding LA size or function.^1^^,^^9^ Thus, patients with isolated LA-CMP are commonly labeled as low-risk patients according to the current decision trees.

Although several studies have demonstrated the association between impaired LAS and LATS and stroke in patients with AF, to our knowledge, none has focused specifically on a low-risk cohort.^10^, ^11^, ^12^, ^13^, ^14^, ^15^ Moreover, most published studies have examined patients in AF and only a few have examined patients in SR.^12^

In this study, we aimed to evaluate the prevalence of LATS in patients deemed low-risk for LAA thrombus by current guidelines, and the association between TTE markers of LA-CMP and presence of LATS.

## Methods

### Study population

We retrospectively included consecutive patients where a TTE and TOE was performed in the same session between July 2020 and December 2023. To control for inter-observer variability in imaging acquisition particularly regarding the LAA, only examinations performed by the same physician (MSR) were included. An extensive database search was conducted from institutional electronic medical records regarding clinical data, medication, and recent medical history. Patients with missing clinical data or poor image quality were excluded from the study.

For this study, we first divided the patients in 2 groups based on the electrocardiogram or, in patients undergoing catheter ablation, based on the rhythm documented on the day of the TOE: (1) patients in SR and (2) patients with atrial arrhythmias. Most patients with atrial arrythmias had either AF or atrial flutter (AFL); for simplicity, this group is referred to as the AF/AFL group throughout the manuscript.

### Low risk for thromboembolic events

On the grounds of the extensive database search regarding each patient, we further identified those patients who were at low risk for the presence of spontaneous echo contrast (SEC), sludge, or thrombus in the LAA according to the current European Society of Cardiology guidelines.^1^

Patients were classified as low risk if they met all the following criteria: adequate anticoagulation for ≥3 weeks prior to TOE if, no history of MS (≥grade 2), no prior stroke or TIA, no previous mitral valve surgery for MS, no known history of LAA thrombus, no history of thromboembolism, no known cardiac amyloidosis, no known hypertrophic cardiomyopathy and no thrombophilia. Patients in SR without anticoagulation if CHA_2_DS_2_-VA = 0 were also classified as low-risk.

This low-risk cohort was then further stratified into 3 subgroups: (1) low-risk patients in SR with a history of AF, (2) low-risk patients currently in AF/AFL, and (3) and low-risk patients without any history of AF.

### Echocardiographic analysis

For this analysis, all TOE examinations were re-assessed by one experienced echocardiographer (AF) for the presence of SEC, sludge, or thrombus in the LA, whereas all TTE examinations were independently re-assessed by 2 experienced echocardiographers (AF, FS), who were blinded to the patients’ clinical data.

LAS analysis was performed offline using 2D-speckle tracking echocardiography (STE) in the apical 4-chamber (A4C) and apical 2-chamber (A2C) views, using the TomTec-Arena TTA.51.4 software, according to current recommendations.^16^, ^17^, ^18^ The average of the A4C and A2C peak longitudinal strain (reservoir strain) values was calculated. In patients with AF, the analysis was performed over 2 consecutive cardiac cycles, and then the mean value was used. Patients with a HR>120 bpm or with poor image quality- defined as non-visualization of ≥1 segment of the LA- were excluded from the strain analysis.

Additionally, bi-plane maximal and minimal LA volume index (LAVImax and LAVImin) was measured according to guideline recommendations.^16^ LA ejection fraction (LA-EF) was calculated via (LAVmax-LAVmin)/LAVmax∗100, using the A4C and A2C views, and the mean was recorded.

**Figure 1:**
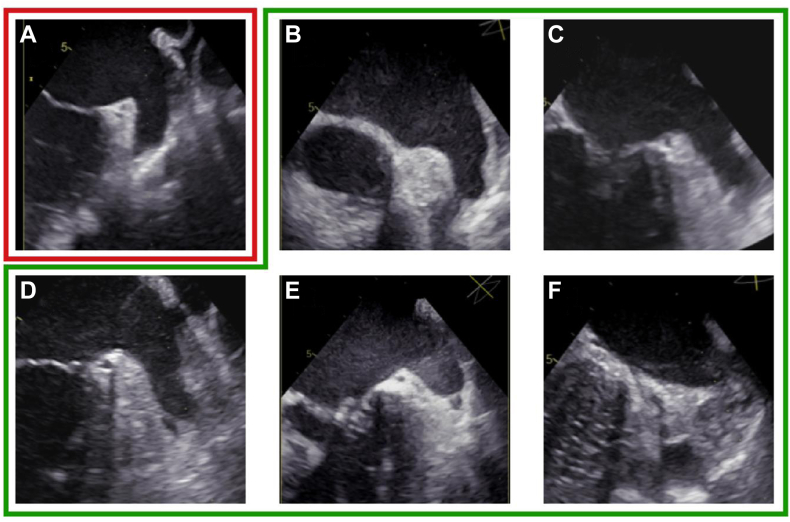
LATS stages. **A:** SEC 1+ : minimal echogenicity, typically confined to the LAA. **B:** SEC 2+ : mild to moderate swirling, detectable without increased gain. **C:** SEC 3+ : moderate, dense swirling, persistent throughout the cardiac cycle. **D:** SEC 4+ : severe echodensity with slow, layered swirling extending into the main LA cavity. **E:** LAA sludge: dense, gelatinous echodensity without a discrete mass, suggestive of pre-thrombotic state **F:** LAA thrombus: organized, well-defined mass. TOE = transesophageal echocardiography; LATS = left atrial thromboembolic substrate; SEC = spontaneous echo contrast.

SEC was graded on a scale from 0 to 4 based on established criteria (Figure 1)^3^^,^^19^:

Clinically relevant findings in the LAA were considered as the presence of SEC ≥2+, sludge or thrombus and were collectively defined as LA LATS. Notably, SEC = 1+ was not considered clinically relevant due to its common occurrence, high interobserver variability and susceptibility to misinterpretation, particularly because it can be influenced by image contrast settings.

### Ethics statement

The research reported in this article adhered to the principles of the Declaration of Helsinki. The study was approved by the institutional ethics committee of Charité (approval number EA1/308/23). The requirement for written informed consent was waived by the ethics committee due to the retrospective nature of the study and the use of fully de-identified data.

### Statistical analysis

Continuous data were presented as mean ± standard deviation and dichotomous data in percentages. Categorical variables were compared by χ^2^ test and Fisher’s exact test. Differences in continuous variables between 2 groups were analyzed using Student’s t-test, whereas comparisons between 3 or more groups were analyzed using a one-way analysis of variance.

The diagnostic performance of LAS and LAVI for predicting the presence of clinically relevant SEC (defined as SEC ≥2+), sludge, or thrombus in the LAA was assessed using receiver operating characteristic (ROC) curve analysis. The area under the curve (AUC) was used to evaluate the discriminatory ability of each parameter. The optimal cut-off value for LAS was determined using the Youden Index (Youden Index = sensitivity + specificity − 1). ROC curves of LAS and LA volumetric parameters (eg, LAVI) were compared using DeLong’s method.

Multivariable logistic regression analysis was performed to identify independent predictors of clinically relevant SEC, sludge, or thrombus in the LAA, adjusting for potential confounding variables. These included the following: CHA_2_DS_2_-VA score, rhythm group, history of stroke/TIA, effective anticoagulation for at least 3 weeks, along with the following functional and anatomical measures of the LA: LAS, LAVImax, LAVImin, and LAEF. Odds ratios (OR) and 95% confidence intervals (CI) were reported for each variable.

Furthermore, we performed binary logistic regression to estimate the probability of LATS using 2 models, and compared their predictive performance with ROC curve analysis.

Model 1 included variables used in current guideline algorithms for TOE before ablation or cardioversion—CHA_2_DS_2_-VA score, history of stroke/TIA, anticoagulation status, and rhythm category—to calculate the baseline predicted probability (p_1_).

Model 2 added LAS to Model 1 to calculate an updated predicted probability (p_2_).

The ROC curves derived from p_1_ and p_2_ were then compared using DeLong’s test to evaluate whether LAS provided incremental diagnostic value beyond the guideline-based model.

All statistical analyses were performed using SPSS version 30.0.0.0 (IBM Corp) and R software (version 4.4.2). A 2-sided *P*-value <0.05 was considered statistically significant.

## Results

After removing duplicates and excluding patients with missing data or poor image quality, a total of 645 patients were included in the final analysis (n = 384 patients in SR, n = 261 patients in AF/AFL). From the entire study cohort, we identified 323 low risk patients for LATS. These patients were further categorized as follows: low-risk patients in SR with a history of AF (n = 105), low-risk patients currently in AF/AFL (n = 157), and low-risk patients in SR without any history of AF (control group, n = 61).

In the overall group, the left ventricle was of normal size with mild hypertrophy and preserved systolic function (mean left ventricular ejection fraction 56%). The mean maximal aortic valve velocity was 2.6 m/s, and at least moderate mitral regurgitation was found in 17% of patients. LA size was moderately increased in patients with AF and markedly higher than in SR controls, accompanied by reduced LA function and strain. Within the low-risk cohort, the prevalence of cardiovascular comorbidities was low, with no prior stroke reported and normal left ventricular dimensions and function. The clinical and echocardiographic characteristics of the different patient groups are summarized in Table 1.

**Table 1:** Clinical and echocardiographic characteristics of the study population

Parameter	All patients		Low-risk patients			*P*-value low-risk patients
	Patients in SR n = 384	Patients in AF/AFL n = 261	Controls n = 61	SR with History of AF n = 105	AF/AFL Patients n = 157	
**Clinical**						
Women, n (%)	147 (38.3)	99 (37.9)	25 (41)	41 (39)	65 (41.4)	.897
Age (years), mean ± SD	60.8 ± 14.8	71.4 ± 10.7	55.3 ± 16.6	66.7 ± 12.4	71.1 ± 10.6	<.001
Height (cm), mean ± SD	178.7 ± 10	176.4 ± 10.8	173.3 ± 8.1	175.8 ± 10.7	173.5 ± 10.5	.352
Weight (kg), mean ± SD	91.6 ± 18.5	86.6 ± 17.5	77 ± 19.9	83.8 ± 17.7	83.7 ± 19.1	.079
BSA (m2), mean ± SD	2.1 ± 1.2	2.1 ± 1	1.9 ± 0.26	2 ± 0.2	2.1 ± 0.3	.045
Heart rate (bpm), mean ± SD	70 ± 13.8	83.6 ± 16.8	73.3 ± 14.7	67 ± 14.2	85.1 ± 17	<.001
Prior stroke, n (%)	131 (34.1)	47 (18)	0	0	0	
Arterial hypertension, n (%)	221 (57.6)	196 (75.1)	16 (26.2)	73 (69.5)	119 (75.8)	<.001
Peripheral artery disease (PAD), n (%)	19 (4.9)	13 (5)	3 (4.9)	3 (2.9)	6 (3.8)	.798
Diabetes mellitus, n (%)	75 (19.5)	63 (24.1)	12 (19.7)	14 (13.3)	32 (20.4)	.338
Coronary artery disease (CAD), n (%)	97 (25.3)	107 (41)	17 (27.9)	34 (32.4)	64 (40.8)	.15
Prior MI, n (%)	22 (5.7)	19 (7.3)	4 (6.6)	5 (4.8)	10 (6.4)	.361
LAA closure, n (%)	10 (2.6)	13 (5.0)	0	3 (2.9)	3 (1.9)	.42
**Medication**						
Anticoagulation, n (%)	139 (36.2)	206 (78.9)	0	96 (91.4)	157 (100)	<.001
- Rivaroxaban, n (%)	35 (9.1)	53 (20.3)	0	28 (26.7)	42 (26.8)	<.001
- Apixaban, n (%)	62 (16.1)	94 (36.0)	0	45 (42.9)	71 (45.2)	<.001
- Edoxaban, n (%)	14 (3.6)	33 (12.6)	0	8 (7.7)	24 (15.3)	<.001
- Dabigatran, n (%)	0	3 (1.2)	0	0	3 (1.9)	.06
- VitK antagonist, n (%)	24 (6.3)	20 (7.7)	0	12 (11.4)	14 (8.9)	.06
- Low molecular volume Heparin, n (%)	4 (1)	3 (1.1)	0	2 (1.9)	2 (1.2)	.86
**Echocardiography**∗						
LVEDD 2D (mm), mean ± SD	46.9 ± 6.4	47 ± 7.3	46 ± 6.9	47.5 ± 6	46.4 ± 6.9	.442
IVSd 2D (mm), mean ± SD	11.2 ± 2.7	11.9 ± 2.4	10.3 ± 2.9	11 ± 2.2	12 ± 2.7	<.001
LVEDV bi-plane (ml), mean ± SD	130.5 ± 44.1	124.3 ± 39.5	131.7 ± 35.7	122 ± 26.8	133.8 ± 39.6	.418
LVESV bi-plane (ml), mean ± SD	60.7 ± 35.4	72.3 ± 39.6	61.0 ± 24.4	58.3 ± 25.2	79.4 ± 40.4	.062
LVEF (%), mean ± SD	58.8 ± 7.9	53.2 ± 11.6	59.2 ± 7.7	57.23 ± 8.1	53.2 ± 11.5	<.001
RA area (cm^2^), mean ± SD	17.3 ± 4.7	24.5 ± 6.9	16.2 ± 3.3	19.4 ± 5.2	22.2 ± 5.3	.011
MV E-Vmax (m/s), mean ± SD	0.76 ± 0.2	0.9 ± 0.2	0.8 ± 0.1	0.8 ± 0.1	0.8 ± 0.1	.294
MV A-Vmax (m/s), mean ± SD	0.71 ± 0.2	N/A	0.6 ± 0.2	0.6 ± 0.2	N/A	.964
AV Vmax (m/s), mean ± SD	2.6 ± 0.5	2.4 ± 0.5	2.7 ± 0.5	2.6 ± 0.5	2.4 ± 0.5	.72
Mitral regurgitation ≥2, n (%)	42 (11)	66 (25.3)	5 (8.2)	24 (22.7)	43 (27.4)	<.001
Mitral stenosis ≥2, n (%)	0	1 (0.4)	0	0	0	
**Left atrial volume and function**						
LAVImax (ml/m^2^), mean ± SD	37.6 ± 17.1	52.5 ± 22.5	36.43 ± 13.7	40.5 ± 14.7	54.1 ± 23.9	<.001
LAVImin (ml/m^2^), mean ± SD	21.0 ± 13.3	41 ± 20.5	18.1 ± 8.5	24.3 ± 12.6	42.3 ± 21.7	<.001
LAEF (%), mean ± SD	47.2 ± 12.7	24.6 ± 11	51.4 ± 10.2	42.2 ± 13.7	23.8 ± 10.7	<.001
LAS (%), mean ± SD	28.7 ± 10.9	10.6 ± 5.5	34.2 ± 11.7	24.7 ± 10	10.2 ± 5.7	<.001

Data are expressed as mean ± SD unless otherwise indicated. LAS refers to the mean of LAS in the apical 4- and apical 2-chamber views and is indicated in absolute values.

AF/AFL= atrial fibrillation/atrial flutter; AV Vmax = peak aortic valve velocity; BSA = body surface area; IVSd 2D = interventricular septal thickness in diastole in 2D imaging; LAA = left atrial appendage; LAS = left atrial strain; LAVImax = maximum left atrial volume index; LAVImin = minimum left atrial volume index; LVEDD 2D = left ventricular end-diastolic diameter in 2D imaging; LVEDV bi-plane = left ventricular end-diastolic volume measured by bipbi-plane method; LVEF = left ventricular ejection fraction; LVESV bi-plane = left ventricular end-systolic volume measured by bipbi-plane method; MV A-Vmax = peak late diastolic mitral inflow velocity (atrial contraction); MV E-Vmax = peak early diastolic mitral inflow velocity; N/A = not applicable; RA area = right atrial area; SR = sinus rhythm.

∗ Echocardiographic variables were calculated using available data; sample size may vary due to missing data.

Indications for TOE were primarily thrombus exclusion prior to catheter ablation or electrical cardioversion (35.7%), suspected endocarditis (18.3%), search for source of embolism (17.1%), evaluation of valvular heart disease (12.3%), and assessment for LAA/patent foramen ovale occluder (3.3%). The study population comprised both ambulatory and hospitalized patients.

### Left atrial thromboembolic substrate findings

After excluding mild spontaneous echocardiographic contrast (SEC 1+) to minimize misclassification, LATS were identified in 106/645 (16.4%) patients from the overall study population (8/384 [2.1%] in SR and 98/261 [37.5%] in AF/AFL).

Among patients classified as low-risk for thromboembolic events (n = 323), LATS was observed in 59 patients (18.3%): 3/105 (2.9%) in SR with a history of AF, 54/157 (34.4%) in AF/AFL, and 2/61 (3.3%) in the control group without any history of AF. Further details are provided in Table 2.

**Table 2:** Left atrial thromboembolic substrate findings in the left atrial appendage

Patient Category	SEC +1	SEC +2	SEC +3	SEC +4	Sludge	Thrombus
All patients SR (n = 384)	8 (2.1%)	4 (1%)	1 (0.3%)	3 (0.8%)	0	0
All patients AF/AFL (n = 261)	34 (13%)	24 (9.2%)	29 (11.1%)	17 (6.5%)	21 (8%)	7 (2.7%)
Low-risk patients SR (n = 105)	7 (6.7%)	1 (1%)	0	2 (1.9%)	0	0
Low-risk patients AF/AFL (n = 157)	28 (17.8%)	16 (10.2%)	13 (8.3%)	11 (7%)	11 (7%)	3 (1.9%)
Controls (n = 61)	0	2 (3.3%)	0	0	0	0

AF/AFL = atrial fibrillation/atrial flutter; SEC = spontaneous echo contrast; SR = sinus rhythm.

### Total study population

We performed a multivariable logistic regression analysis to identify predictors of LATS. After adjusting for effective anticoagulation (OR: 1.132; *P =* .784), CHA_2_DS_2_-VA score (OR: 1.181; *P =* .176), rhythm group (OR: 2.105; *P =* .146), prior stroke or TIA (OR: 1.111; *P =* .838), LAVImax (OR: 0.975; *P =* .708), LAVImin (OR: 1.036; *P =* .665), and LAEF (OR: 0.991; *P =* .847), only LAS remained statistically significant and independently predicted LATS (OR: 0.819; *P <* .001; n = 481). This indicates that each 1% increase in LAS was associated with approximately an 18% reduction in the odds of LATS. None of the other clinical or echocardiographic parameters in the model demonstrated statistically significant associations.

**Figure 2:**
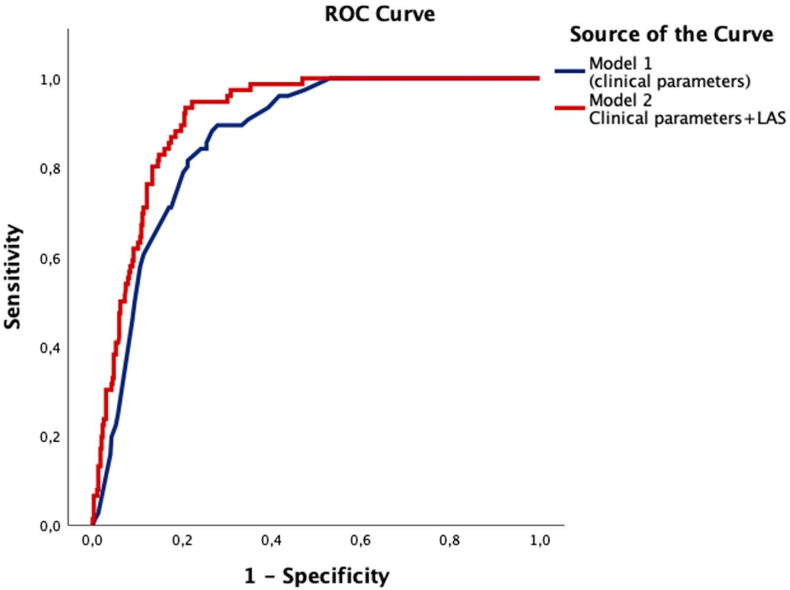
Receiver operating characteristic (ROC) curves for association with left atrial appendage thromboembolic substrate. Model 1, based on current guideline-recommended variables including rhythm group, CHA_2_DS_2_-VA score, anticoagulation status, and prior stroke or TIA, demonstrated good discriminative ability (AUC = 0.859; 95% CI: 0.828–0.899; *P <* .001). The addition of left atrial strain (LAS) in Model 2 significantly improved prediction accuracy, achieving excellent discrimination (AUC = 0.906; 95% CI: 0.880–0.935; n = 481; *P <* .001), with the difference between models being statistically significant (DeLong test, *P <* .001). ROC = receiver operating characteristic; TOE = transesophageal echocardiography; LAS = left atrial strain; AUC = area under the curve; CI = confidence interval; TIA = transient ischemic attack.

We further assessed the predictive performance of 2 multivariable models using ROC curve analysis. Model 1 comprised clinical parameters routinely assessed when determining the need for pre-procedural transesophageal echocardiography according to current guideline recommendations, including CHA_2_DS_2_-VA score, rhythm category, history of prior stroke or TIA, and anticoagulation status. Model 2 incorporated LAS in addition to the variables in Model 1. The AUC for Model 1 was 0.859 (SE = 0.018; 95% CI: 0.828–0.899; *P <* .001), whereas Model 2 achieved an AUC of 0.906 (SE = 0.014; 95% CI: 0.880–0.935; *P <* .001). Comparison of the 2 ROC curves using DeLong’s test demonstrated that the inclusion of LAS significantly improved discriminatory ability (*P <* .001) (Figure 2).

### Sinus rhythm

**Figure 3:**
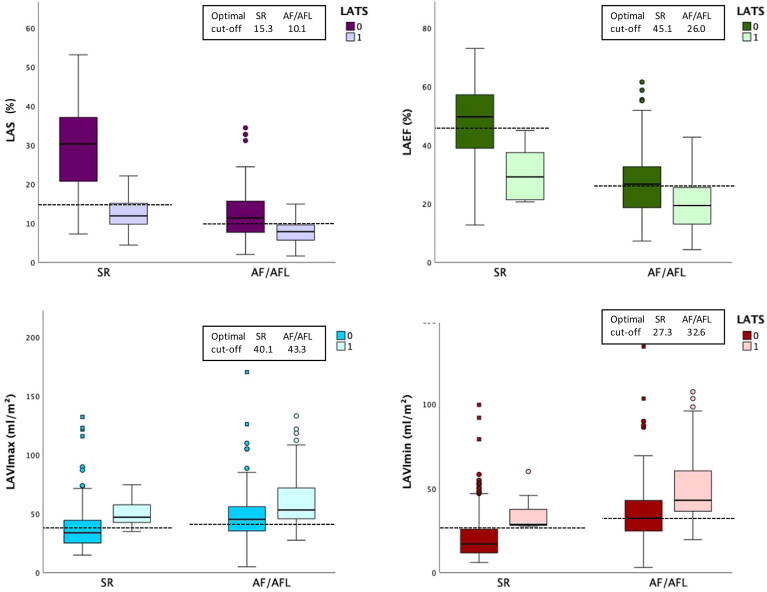
Left atrial functional parameters according to LATS findings, stratified by rhythm. Box plots show LAS, LAEF, LAVImax, and LAVImin in patients with and without LATS (defined as SEC ≥ 2+, sludge, or thrombus), stratified by cardiac rhythm. In SR, presence of LATS was associated with significantly lower LAS (12.5% vs 29.2%, p < 0.001; n = 304), lower LAEF (30.2% vs 47.6%, p < 0.001; n = 305), higher LAVImax (50.9 vs 37.3 mL/m^2^, p = 0.037; n = 292), and higher LAVImin (35.3 vs 20.6 mL/m^2^, p = 0.004; n = 287). Similarly, in AF/AFL, LATS was associated with significantly lower LAS (7.9% vs 12.2%, p < 0.001; n = 183), lower LAEF (20.0% vs 27.3%, p < 0.001; n = 219), and higher LAVImax (60.1 vs 48.4 mL/m^2^, p < 0.001; n = 216) and LAVImin (49.3 vs 36.0 mL/m^2^, p < 0.001; n = 210). *Boxes* represent interquartile ranges with median lines; *whiskers* indicate 1.5× IQR; outliers are shown as *dots*. Optimal cut-offs are shown by *dashed lines*. Patients with LATS demonstrated impaired left atrial function and increased atrial volumes. AF = atrial fibrillation; AFL = atrial flutter; LAEF = left atrial emptying fraction; LAS = left atrial strain; LATS = left atrial thromboembolic substrate; LAVImax = maximum left atrial volume index; LAVImin = minimum left atrial volume index; SEC = spontaneous echo contrast; SR = sinus rhythm.

In patients with SR, the presence of LATS was associated with markedly impaired LA function and increased atrial volumes. Patients with LATS demonstrated substantially lower LAS and LAEF, alongside higher LAVImax and LAVImin (Figure 3).

ROC analysis in patients with SR indicated that LAS has the highest diagnostic accuracy for LATS (AUC: 0.909, 95% CI: 0.839–0.978, *P* < 0.001), outperforming LAVImin (AUC: 0.844), LAEF (0.847), and LAVImax (0.783). Optimal cut-offs were: LAS ≤ 15.3% (sensitivity 86%, specificity 89%), LAVImin ≥ 27.3 mL/m^2^ (100%, 78%), LAEF ≤ 45.1% (100%, 61%), and LAVImax ≥ 40.1 mL/m^2^ (86%, 68%).

### AF/AFL cohort

Similarly, in the AF/AFL cohort, patients with LATS were consistently associated with reduced LA functional parameters and increased atrial volumes (Figure 3).

ROC analysis showed that LAS had the highest diagnostic accuracy among LA parameters, with an AUC of 0.754 (95% CI: 0.684–0.825, *P* < 0.001), outperforming LAVImin (AUC: 0.704), LAEF (0.705), and LAVImax (0.659). The optimal LAS cut-off was ≤10.1%, yielding a sensitivity of 88.9% and specificity of 60%. For LAVImax, a cut-off of ≥43.3 mL/m^2^ showed moderate accuracy with a sensitivity of 85.7% and specificity of 43.6%.

### Low-risk patients for left atrial thromboembolic substrate

#### Sinus rhythm

**Figure 4:**
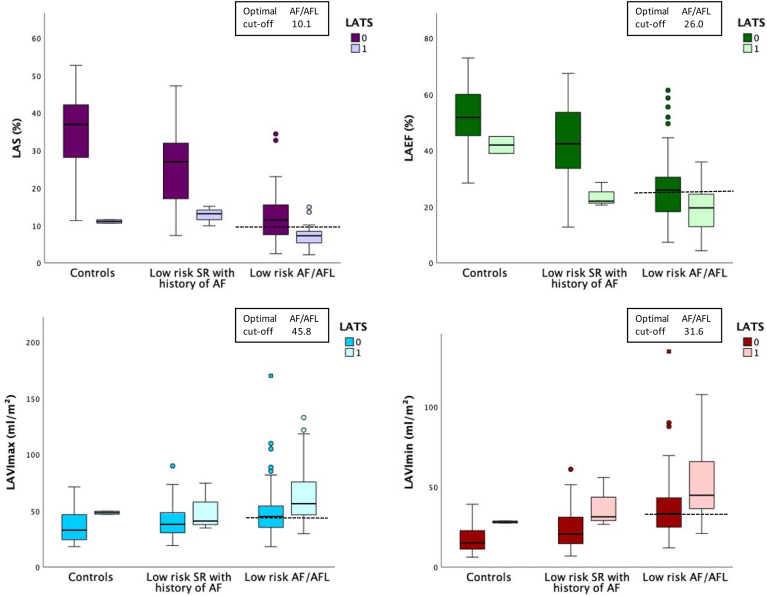
Left atrial functional parameters according to LATS findings in patients with low thromboembolic risk. Box plots show LAS, LAEF, LAVImax, and LAVImin in patients with and without LATS; defined as SEC ≥2+, sludge, or thrombus, stratified by patient group: controls (SR without AF history), low-risk SR with history of AF, and low-risk AF/AFL. In controls (SR without AF history, total n = 62), patients with LATS (n = 2) had significantly lower LAS compared with those without LATS (11.1% vs 35.3%, *P =* .003; LAS available in 46 patients). No significant differences were observed in LAEF (41.9% vs 51.9%, *P =* .178; n = 46), LAVImax (48.3 vs 35.8 mL/m^2^, *P =* .210; n = 45), or LAVImin (28.0 vs 17.6 mL/m^2^, *P =* .092; n = 45). In low-risk patients with history of AF in sinus rhythm (total n = 105), LATS-positive patients (n = 3) demonstrated significantly lower LAS (12.7% vs 25.1%, *P =* .033; LAS available in 87 patients) and significantly lower LAEF (23.7% vs 43.0%, *P =* .016; n = 89) compared with LATS-negative patients. Differences in LAVImax (50.1 vs 40.2 mL/m^2^, *P =* .255; n = 82) and LAVImin (37.8 vs 23.8 mL/m^2^, *P =* .058; n = 82) did not reach statistical significance. In low-risk AF/AFL patients, LATS was associated with significantly lower LAS (7.1% vs 12.0%, p < 0.001; n = 101), lower LAEF (19.2% vs 26.1%, *P* < 0.001; n = 134), and higher LAVImax (64.0 vs 48.8 mL/m^2^, *P* < 0.001; n = 132) and LAVImin (52.4 vs 36.9 mL/m^2^, *P* < 0.001; n = 131). *Boxes* represent interquartile ranges with median lines; *whiskers* indicate 1.5 × IQR; outliers are shown as *dots*. Optimal cut-offs are shown by *dashed lines*. Across all groups, patients with LATS demonstrated impaired left atrial function and increased atrial volumes. AF = atrial fibrillation; AFL = atrial flutter; LAEF = left atrial emptying fraction; LAS = left atrial strain; LATS = left atrial thromboembolic substrate; LAVImax = maximum left atrial volume index; LAVImin = minimum left atrial volume index; SEC = spontaneous echo contrast; SR = sinus rhythm.

In low-risk patients with a history of AF currently in SR, those with LATS demonstrated significantly lower LAS values (24.1 ± 9.9%) compared with those without LATS (12.7 ± 2.6%, p = 0.033) (Figure 4). Among low-risk patients in SR without a history of AF, LAS values were also significantly lower in those with LATS (11.1 ± 0.6% vs 33.5 ± 10.8%, *P* = 0.003).

ROC analysis was not performed in the low-risk SR subgroup with prior AF due to the small number of LATS cases (n = 3) (Figure 4).

#### AF/AFL cohort

In low-risk patients with AF or AFL, all parameters of LA structure and function were significantly associated with the presence of LATS. LAS was significantly lower in patients with LATS, whereas LAVImax and LAVImin were significantly higher. LAEF was also significantly reduced in patients with LATS (Figure 4).

The discriminatory ability of LAS in this group was moderate, with an AUC of 0.762. For comparison, LAVImin, LAEF, and LAVImax had AUCs of 0.715, 0.706, and 0.684, respectively. The optimal cut-off for LAS was 10.1% yielding a sensitivity of 95% and a specificity of 58%.

Table 3 summarizes how well the optimal LAS cut-offs of our study discriminate between LATS and no LATS in low-risk patients.

**Table 3:** Presence of LATS in the low-risk cohort

Subgroup				
**Low-risk in AF/AFL**		LAS <10.1	LAS ≥10.1	*P*-value
*(cut-off 10.1%)*	Total, n (%)	61 (60.4%)	40 (39.6%)	<.001
	LATS present, n (%)	35 (57.4%)	3 (7.5%)	
**Low-risk in SR with AF history**		LAS <15.3	LAS ≥15.3	*P*-value
*(cut-off 15.3%)*	Total, n (%)	20 (23.0%)	67 (77.0%)	.011
	LATS present, n (%)	3 (15.0%)	0 (0%)	
**Low-risk in SR without AF history***(cut-off 15.3%)*		LAS <15.3	LAS ≥15.3	*P*-value
	Total, n (%)	3 (6.5%)	43 (93.5%)	.003
	LATS present, n (%)	2 (66.7%)	0 (0%)	

Distribution of patients according to left atrial strain cut-off values within predefined low-risk subgroups: (1) AF/AFL (2) SR with prior AF history, and (3) SR with no history of AF. For each subgroup, the number and percentage of patients below and above the LAS cut-off are shown, along with the proportion of patients with LATS within each LAS stratum. The *P*-values indicate the statistical significance of the difference in LATS prevalence between LAS strata.

AF/AFL = atrial fibrillation/atrial flutter; LATS = left atrial thromboembolic substrate; SR = sinus rhythm.

### Inter-observer variability

Inter-observer variability was assessed in a random subset of 10 patients. Measurements of LAVmax, LAVmin (A4C and A2C views), and LAS (A4C and A2C) were performed independently by 2 experienced observers blinded to each other’s results. Agreement was evaluated using the intraclass correlation coefficient (ICC, 2-way random effects model, absolute agreement, single measures). Inter-observer reproducibility was excellent for all variables, with ICCs ranging from 0.982 to 0.999. ICCs were 0.998 for LAVmax A4C, 0.999 for LAVmin A4C, 0.994 for LAVmax A2C, 0.998 for LAVmin A2C, 0.982 for LAS A4C, and 0.992 for LAS A2C.

## Discussion

Our study reveals frequent presence of LATS in patients deemed low-risk according to current guidelines, particularly in those with AF. Across all rhythm categories, LAS consistently showed strong inverse association with the presence of LATS, proving a robust transthoracic echocardiographic marker for identifying these patients. Furthermore, comparison of LAS with conventional volumetric echocardiographic parameters of the left atrium to predict LATS demonstrated a clear superiority of LAS, confirming previous findings of LA dysfunction even when LA size is within normal limits.^8^

### Patients in sinus rhythm

Concordant to previously published data, presence of LATS was rare in low-risk patients with SR.^3^ In the cases where LATS was evident, we observed a clear difference in the distribution of LAS between those with and without LATS.

The presence of LATS even in SR patients without a known history of AF underscores the heterogeneity of LA pathology and the potential role of subclinical atrial disease. Numerous studies have identified LAS as a surrogate marker for detecting previously undiagnosed paroxysmal AF in cryptogenic stroke patients.^10^^,^^11^^,^^20^^,^^21^ Additionally, these results may support the notion that thromboembolic risk is not exclusively rhythm-dependent but is also mediated by atrial structure and functionality.

### Patients in AF/AFL

Previous studies have reported LAA thrombus even in anticoagulated patients with AF.^12^, ^13^, ^14^^,^^22^^,^^23^ Others have demonstrated associations between impaired LAS and LAA thrombus or SEC in patients with AF.^10^^,^^11^^,^^24^^,^^25^ To our knowledge, our study is the first to focus on LAS performance in patients meeting clinical low-risk criteria. In this low-risk cohort, 34.4% of patients had LATS, 8.9% had sludge or LAA thrombus. In concordance with the SR patients, LAS was able to discriminate between those with and without LATS.

LAS is known to be significantly reduced in AF patients. Additionally, technical challenges in AFs—including elevated heart rate and cycle-to-cycle variability—should be considered. Consequently, the cut-offs in SR cannot be applied to AF/AFL patients. In our data, the optimal LAS threshold was 10.1%, with a sensitivity of 95% and specificity of 61%. Notably, 2 studies conducted during the COVID-19 pandemic in patients with AF scheduled for elective cardioversion (eCV) reported similar findings, identifying an LAS cut-off of 9% to identify the presence of LAA thrombus.^26^^,^^27^

### Thromboembolic risk stratification prior to rhythm control in AF

Current guidelines recommend visualization of the LAA prior to cardioversion in patients with new-onset AF of >24 hours’ duration who are not adequately anticoagulated, although it is acknowledged that the exact time of AF onset is often difficult to define.^1^ In contrast, there is no universal recommendation regarding routine LAA thrombus exclusion before AF ablation. Two meta-analyses have reported a prevalence of LAA thrombus of 1.3% and 2.7% in patients scheduled for AF ablation despite adequate anticoagulation.^22^^,^^23^ Data on the prevalence of dense SEC or sludge are limited, as most studies focus exclusively on overt thrombus; nevertheless, the presence of these findings frequently leads to postponement of ablation.

Certain high-risk patient groups—including those with cardiac amyloidosis, rheumatic heart disease, hypertrophic cardiomyopathy (HCM), prior stroke, or prior LAA thrombus—remain at increased risk for LAA thrombus despite adequate anticoagulation. In addition, patients with non-paroxysmal AF have a higher thrombotic risk compared with those with paroxysmal AF.^1^ Conversely, in selected low-risk patients receiving adequate anticoagulation, TOE may be omitted. However, these strategies are largely based on observational data, as randomized controlled trials are lacking.^1^ Consequently, individual centers rely on local protocols for LAA visualization prior to AF ablation, underscoring the need for standardized and universally applicable approaches.

Our findings support integrating LAS into this decision-making framework. This approach aligns with accumulating evidence that LA mechanical dysfunction—rather than clinical risk scores alone—represents a key factor for thrombogenesis. Multiple studies have demonstrated that LA reservoir strain independently predicts stroke risk and LAA thrombus beyond CHA_2_DS_2_-VASc scoring, with excellent discriminatory capacity in patients undergoing TOE.^28^, ^29^, ^30^, ^31^

In this context, multivariable logistic regression analysis identified LAS as the only independent predictor of LA thrombus or sludge (LATS) in our cohort. Importantly, adding LAS to a clinical model comprising CHA_2_DS_2_-VA score, heart rhythm, and prior stroke significantly improved discrimination for LATS (AUC 0.906 vs 0.859 without LAS). By comparison, a recent study proposed a score based on clinical and basic echocardiographic parameters without incorporating LA functional indices and reported a more modest AUC of 0.780 for predicting LAA thrombus.^32^

Given the well-established association between LATS and future embolic events, our results call for a re-evaluation of current risk stratification algorithms, particularly when determining the need for TOE before procedures such as cardioversion or AF ablation. Incorporating transthoracic assessment of LA functional and volumetric parameters may help identify a subgroup of patients who would benefit from additional TOE imaging despite appearing low-risk according to existing clinical decision pathways.

Despite this promise, several barriers currently limit widespread clinical implementation. The recent consensus statement of the American Society of Echocardiography and the European Association of Cardiovascular Imaging highlights the predictive value of LA strain but emphasizes that specific cut-off values for clinical decision-making remain investigational, and that vendor-related discrepancies in strain measurements must be considered.^17^ Addressing these gaps will be essential before LAS can be routinely integrated into standardized preprocedural workflows.

### Future research

Due to the retrospective nature of this investigation, several questions remain unanswered and should be addressed in further research. Future prospective studies should evaluate whether LAS-guided risk stratification improves clinical decision-making, reduces thromboembolic events, or safely limits the need for TOE before rhythm-control procedures compared with current guideline-based strategies. Given the high number of patients labeled low-risk with AF/AFL and presence of LATS, prospective studies should investigate if presence of pronounced LA-CMP may lead to particularly low flow in the LAA, possibly not sufficiently addressable by oral anticoagulation. Patients with pronounced LA-CMP might be a subgroup which predominantly benefits from early LAA occluder implantation. Moreover, the impact of different direct oral anticoagulants and the potential benefit of switching agents warrant investigation. Finally, the clinical relevance and particularly the acute thromboembolic potential of SEC is unknown.

## Limitations

This study has several limitations. Its retrospective and single-center design may limit generalizability. LAS measurements were not feasible in all patients due to image quality constraints, particularly in those with high heart rates or poor acoustic windows. Furthermore, in the low-risk subgroup in SR, the number of patients with LATS was relatively small (n = 5), which limits the statistical power for subgroup analyses and may affect the robustness of conclusions in this population. Accordingly, the LAS cut-off values derived from these subgroup analyses should be interpreted with caution and considered exploratory rather than definitive thresholds for clinical decision-making. Additionally, CHA_2_DS_2_-VA score was originally intended for prediction of stroke risk and not for the presence of LATS.

The association of spontaneous echo contrast and sludge with stroke risk is not well studied. Although LAS was shown to be a strong predictor of LATS, the cross-sectional nature of the study does not allow assessment of actual thromboembolic events (eg, stroke or TIA). Longitudinal follow-up would be necessary to confirm the prognostic value of LAS. Finally, although the low risk cohort included only patients with adequate anticoagulation according to guideline definitions—specifically, those in atrial fibrillation with anticoagulation for at least 3 weeks, and those in SR with anticoagulation for at least 3 weeks if their CHA_2_DS_2_-VA score was ≥1—adherence to anticoagulation regimens could not be independently verified in all cases, and due to the retrospective character of our study, poor adherence may have been missed in some of the study patients.

## Conclusion

In this low-risk cohort, 34.4% of AF/AFL patients had at least significant spontaneous echo contrast, 8.9% had sludge or LAA thrombus. LAS showed excellent accuracy predicting LATS. These findings suggest that relying solely on currently established clinical risk scores may overlook a subset of patients at risk for thromboembolic events. Evaluation regarding presence of LA cardiomyopathy via TTE could serve as a valuable adjunct to guide individualized decisions regarding the need for TOE before elective interventions aiming at rhythm control.

## Disclosures

The authors have no conflicts of interest to disclose.
